# Supplementary material for: Relationships between Physical Activity Frequency and Self-Perceived Health, Self-Reported Depression, and Depressive Symptoms in Spanish Older Adults with Diabetes: A Cross-Sectional Study
Source: Int J Environ Res Public Health. 2023 Feb 6;20(4):2857. doi: 10.3390/ijerph20042857 (PMC9958756; doi:10.3390/ijerph20042857)
Supplement: Supplementary file 1 [file ijerph-20-02857-s001.zip › ijerph-2141546-supplementary.pdf]

Supplementary material

Table S1. Linear regression for Depression Symptoms.

EHIS 2014							
Explanatory variable	Unstandardized Coefficients				Beta	t	Sig.
	β	95% CI for β		Std. Error			
(Constant)	1.291	1.161	1.421	0.066		19.421	<0.001
PAF	-0.174	-0.217	-0.131	0.022	-0.216	-7.872	<0.001
Sex	0.241	0.177	0.305	0.033	0.202	7.378	<0.001
**EHIS 2020**							
Explanatory variable	Unstandardized Coefficients				Beta	t	Sig.
	β	95% CI for β		Std. Error			
(Constant)	1.341	1.275	1.407	0.034		39.795	<0.001
PAF	-0.083	-0.113	-0.052	0.016	-0.140	-5.326	<0.001
Sex	0.134	0.081	0.186	0.027	0.131	4.991	<0.001

EHIS: European Health Survey Spain; B: Beta (Standardized coefficients); CI: Confidence interval; Std. Error: Standard error; t: t statistic; Sig: Significance; PAF: Physical Activity Frequency; Sex (Men=0; Women=1).

Table S2. Linear regression for Self-perceived Health

EHIS 2014							
Explanatory variable	Unstandardized Coefficients				Beta	t	Sig.
	β	95% CI for β		Std. Error			
(Constant)	1.886	1.719	2.052	0.085		22.278	0.000
PAF	0.299	0.244	0.355	0.028	0.289	10.619	0.000
Sex	-0.175	-0.257	-0.094	0.042	-0.115	-4.218	0.000
**EHIS 2020**							
Explanatory variable	Unstandardized Coefficients				Beta	t	Sig.
	β	95% CI for β		Std. Error			
(Constant)	2.683	2.411	2.956	0.139		19.299	0.000
PAF	0.146	0.101	0.191	0.023	0.166	6.361	0.000
Sex	-0.171	-0.248	-0.094	0.039	-0.114	-4.360	0.000
BMI	-0.015	-0.023	-0.007	0.004	-0.097	-3.719	0.000

EHIS, European Health Survey Spain; β, Beta (Standardized coefficients); CI, Confidence interval; Std. Error, Standard error; t, t statistic; Sig, Significance; PAF: Physical Activity Frequency; Sex (Men=0; Women=1).

Table S3. Educational level of the participants: EHSS2014

			Sex		
			Men	Women	Overall
Educational Level	Cannot read or write	n	9_a	46_b	55
		%	1.3%	7.3%	4.2%
	Incomplete primary education (Attended less than 5 years of primary school)	n	111_a	191_b	302
		%	16.2%	30.2%	22.9%
	Completed primary education	n	254_a	249_a	503
		%	37.0%	39.4%	38.1%
	First stage of Secondary Education, with or without a diploma (2nd year of secondary school)	n	112_a	69_b	181
		%	16.3%	10.9%	13.7%
	Post-secondary education	n	64_a	25_b	89
		%	9.3%	4.0%	6.7%
	Intermediate professional education or equivalent	n	22_a	12_a	34
		%	3.2%	1.9%	2.6%
	Higher professional education or equivalent	n	33_a	6_b	39
		%	4.8%	0.9%	3.0%
	University studies or equivalent	n	82_a	34_b	116
		%	11.9%	5.4%	8.8%
Overall		n	687	632	1319
		%	100.0%	100.0%	100.0%

n (participantes); % (percentage); Each subscript letter denotes a subset of Sex categories whose column proportions do not differ significantly from each other at the .05 level.

Table S4. Educational level of the participants: EHSS2020

			Sex		
			Men	Women	Overall
Educational Level	Cannot read or write	n	3_a	16_b	19
		%	15.8%	84.2%	100.0%
	Incomplete primary education (Attended less than 5 years of primary school)	n	109_a	148_b	257
		%	42.4%	57.6%	100.0%
	Completed primary education	n	243_a	231_a	474
		%	51.3%	48.7%	100.0%
	First stage of Secondary Education, with or without a diploma (2nd year of secondary school)	n	188_a	140_a	328
		%	57.3%	42.7%	100.0%
	Post-secondary education	n	84_a	45_b	129
		%	65.1%	34.9%	100.0%
	Intermediate professional education or equivalent	n	36_a	29_a	65
		%	55.4%	44.6%	100.0%
	Higher professional education or equivalent	n	41_a	15_b	56
		%	73.2%	26.8%	100.0%
	University studies or equivalent	n	95_a	57_b	152
		%	62.5%	37.5%	100.0%
Overall		n	799	681	1480
		%	54.0%	46.0%	100.0%

n (participantes); % (percentage); Each subscript letter denotes a subset of Sex categories whose column proportions do not differ significantly from each other at the .05 level.
